# Non-communicable airway disease and air pollution in three African Countries: Benin, Cameroon and The Gambia

**DOI:** 10.5588/ijtldopen.23.0490

**Published:** 2024-04-01

**Authors:** B. Awokola, H. Lawin, O. Johnson, A. Humphrey, D. Nzogo, L. Zubar, G. Okello, S. Semple, E. Awokola, G. Amusa, N. Mohammed, C. Jewell, A. Erhart, K. Mortimer, G. Devereux, B.H. Mbatchou-Ngahane

**Affiliations:** ^1^ Department of Clinical Sciences, Liverpool School of Tropical Medicine, Liverpool, UK;; ^2^ Medical Research Council Unit The Gambia at the London School of Hygiene and Tropical Medicine, Fajara, The Gambia;; ^3^ Occupational Health Unit, University of Abomey Calavi, Abomey-Calavi, Benin Republic;; ^4^ Department of Mathematics, University of Manchester, Manchester, UK;; ^5^ Department of Internal Medicine, Douala General Hospital, Douala,; ^6^ Faculty of Medicine and Pharmaceutical Sciences, University of Douala, Douala, Cameroon;; ^7^ Education for Health Africa, Cape Town, South Africa;; ^8^ Prince of Wales Institute for Leadership Sustainability, University of Cambridge, Cambridge,; ^9^ Institute for Social Marketing and Health, University of Stirling, Stirling, Scotland, UK;; ^10^ Department of Nursing, American International University of West Africa, The Gambia;; ^11^ Department of Internal Medicine, Jos University Teaching Hospital, Jos,; ^12^ Department of Medicine, University of Jos, Jos, Nigeria;; ^13^ Centre for Health Informatics, Computing and Statistics (CHICAS), Lancaster University, Lancaster,; ^14^ Cambridge Africa, Department of Pathology, University of Cambridge, Cambridge, UK;; ^15^ School of Clinical Medicine, College of Health Sciences, University of KwaZulu-Natal, Durban, South Africa;; ^16^ Liverpool University Hospitals NHS Foundation Trust, Liverpool, UK

**Keywords:** chronic airway disease, pollution, sub-Saharan Africa, CAD, particulate matter

## Abstract

**BACKGROUND:**

Air pollution exposure can increase the risk of development and exacerbation of chronic airway disease (CAD). We set out to assess CAD patients in Benin, Cameroon and The Gambia and to compare their measured exposures to air pollution.

**METHODOLOGY:**

We recruited patients with a diagnosis of CAD from four clinics in the three countries. We collected epidemiological, spirometric and home air pollution data.

**RESULTS:**

Of the 98 adults recruited, 56 were men; the mean age was 51.6 years (standard deviation ±17.5). Most (69%) patients resided in cities and ever smoking was highest in Cameroon (23.0%). Cough, wheeze and shortness of breath were reported across the countries. A diagnosis of asthma was present in 74.0%; 16.3% had chronic obstructive pulmonary disease and 4.1% had chronic bronchitis. Prevalence of airflow obstruction was respectively 77.1%, 54.0% and 64.0% in Benin, Cameroon, and Gambia. Across the sites, 18.0% reported >5 exacerbations. The median home particulate matter less than 2.5 μm in diameter (PM_2.5_) was respectively 13.0 μg/m^3^, 5.0 μg/m^3^ and 4.4 μg/m^3^. The median home carbon monoxide (CO) exposures were respectively 1.6 parts per million (ppm), 0.3 ppm and 0.4 ppm. Home PM_2.5_ differed significantly between the three countries (*P* < 0.001) while home CO did not.

**CONCLUSION:**

Based on these results, preventive programmes should focus on ensuring proper spirometric diagnosis, good disease control and reduction in air pollution exposure.

## RÉSUMÉ

**CONTEXTE:**

L'exposition à la pollution de l'air peut accroître le risque de développement et d'aggravation des maladies chroniques des voies respiratoires (CAD). Nous avons entrepris d'évaluer les patients atteints de CAD au Bénin, au Cameroun et en Gambie et de comparer les niveaux d'exposition à la pollution de l'air qu'ils ont subis.

**MÉTHODOLOGIE:**

Nous avons sélectionné des patients ayant reçu un diagnostic de CAD dans quatre cliniques de ces trois pays. Nous avons collecté des informations épidémiologiques, des mesures spirométriques ainsi que des données sur la pollution de l'air à leur domicile.

**RÉSULTATS:**

En total, 98 individus adultes ont été sélectionnés pour cette étude. Parmi eux, 56 étaient de sexe masculin. L'âge moyen de ces participants était de 51,6 ans, avec un écart-type de ±17,5. La majorité des patients (69%) résidaient en milieu urbain, tandis que le taux de tabagisme le plus élevé était observé au Cameroun (23,0%). Les symptômes de toux, de respiration sifflante et d'essoufflement ont été rapportés dans tous les pays. Parmi les patients, 74% ont reçu un diagnostic d'asthme, 16,3% souffraient de maladie pulmonaire obstructive chronique et 4,1% de bronchite chronique. L'obstruction des voies respiratoires était présente respectivement chez 77,1%, 54,0% et 64,0% des cas au Bénin, au Cameroun et en Gambie. Sur l'ensemble des sites, 18,0% ont signalé plus de cinq exacerbations. La médiane des PM_2.5_ à domicile était de 13,0 µg/m^3^, 5,0 µg/m^3^ et 4,4 µg/m^3^, respectivement. Les expositions médianes au monoxyde de carbone (CO) à domicile étaient de 1,6 ppm, 0,3 ppm et 0,4 ppm respectivement. Les PM_2,5_ à domicile présentaient des différences significatives entre les trois pays (*P* < 0,001), contrairement au CO à domicile.

**CONCLUSION:**

En se basant sur ces résultats, il est recommandé que les programmes de prévention se focalisent sur un dépistage spirométrique adéquat, une gestion efficace de la maladie et une diminution de l'exposition à la pollution atmosphérique.

Asthma is a highly prevalent chronic respiratory disease affecting 4.4% of adults worldwide, which translates to about 343 million people in 2020.^1^ Using the Global Initiative for Chronic Obstructive Lung Disease (GOLD) definition, 212.3 million people had chronic obstructive pulmonary disease (COPD) worldwide in 2019.^2^ From 1990 to 2019, the global prevalence of COPD decreased by 8.7%. During the same period, the number of deaths due to COPD had decreased by 41.7%. However, most of the deaths occurred in low-and-middle income countries (LMICs).^2^ Less is known about the prevalence of asthma and COPD in adults living in sub-Saharan African countries but, in a biomass exposed population in rural Cameroon, the prevalence of COPD was 18.4%,^3^ while the prevalence of adult asthma in Cameroon was 3.9%.^4^ In Benin, a recent study reported an asthma prevalence of 4.1% in the general population.^5^ Air pollution is an important driver of non-communicable diseases such as stroke, lung cancer, COPD and asthma. It is a major public health concern worldwide. Besides smoking, exposure to occupational pollution, biomass cooking and heating are other important risk factors for COPD.^6^ Some studies also suggest air pollution contributes to asthma causation.^7^

Furthermore, the WHO estimates that around 7 million people die every year from exposure to fine particles in polluted air.^8,9^ Global mortality due to ambient particulate matter less than 2.5 μm in diameter (PM_2.5_) increased from 1990 to 2015. Attributable deaths rose from 3·5 million in 1990 to 4·2 million in 2015, representing 7.6% of total global deaths, with ambient PM_2.5_ being the fifth-ranking mortality risk factor. Air pollution has been reported to exacerbate existing CAD, leading to increase of admissions in the emergency department.^10,11^

Some studies have assessed the outdoor air quality index (AQI) in the living area of patients with CAD in order to help them to regulate their daily activity according to the local AQI report and help prevent exacerbation.^11^ In sub-Saharan Africa, studies assessing air pollution in patients with non-communicable chronic airway diseases such as asthma and COPD are scarce. We present here one of the first studies to have related air pollution exposures in people with CAD in three sub-Saharan countries in West Africa.

## METHODOLOGY

### Study design and setting

The study was a multicentre clinic-based cross-sectional study that recruited from four clinics in three sub-Saharan African countries: Benin, Cameroon and Gambia.

### Study setting

Patients were recruited from 1) National Teaching Hospital of Pulmonology, Cotonou, Benin, a tertiary care and teaching centre; 2) Douala General Hospital, Cameroon, a tertiary care and teaching centre; 3) Kanifing General Hospital, The Gambia, a secondary care hospital; and 4) Fajara site of the Medical Research Council Gambia at London School of Hygiene & Tropical Medicine, a tertiary facility serving the country.

Benin is a West African country of 115,000 km^2^ with a population of 13 million and a gross domestic product (GDP) of $11.4 billion.^12^ The population prevalence of COPD from the Benin BOLD study was 7.0%.^13^ Cameroon is a west-central African country bordering the Gulf of Guinea with a GDP of US$44.8 billion and a population of 29.3 million.^14^ Reported asthma prevalence in Cameroon is 3.9%^15^ and reported COPD prevalence ranges from 2.4% to 18.4%.^3,11,16^ The Gambia is the smallest country in mainland Africa (10,689 km^2^), with a population of 1,900,000.^16^ There is paucity of CAD data. Prior to this study, none of these clinics had routine spirometry services.

During a 13-month period (August 2019 to March 2020 in Benin and Cameroon, November 2020 to April 2021 in Gambia), outpatients aged ≥18 years with a physician diagnosed chronic airway disease (asthma, COPD, chronic bronchitis) were recruited consecutively.

### Data collection and quality control

An interviewer administered a respiratory questionnaire based on the Burden of Obstructive Lung Disease (BOLD) questionnaire, with additional questions developed as part of the National Institute for Health Research International Multidisciplinary Programme to Address Lung Health and TB in Africa ‘‘IMPALA’’ Lung health across the Life Course (LuLi) initiative.^17^ The asthma control test (ACT) and the COPD Assessment Test (CAT) were also administered as appropriate.^17–19^

### Spirometry

This was performed according to the Pan African Thoracic Society (PATS), American Thoracic Society (ATS)/European Respiratory Society (ERS) standards by trained and PATS certified technicians.^20^ The EasyOne^®^ spirometer (ndd, Zürich, Switzerland) with a daily calibration check was used. The procedure included at least three acceptable and repeatable forced vital capacity manoeuvres out of up to eight performed while sitting. Inhaled salbutamol 400 µg was administered via a spacer device and post-bronchodilator (BD) blows executed 15 min later. Manual overreading of all the traces was performed. Spirometric outcomes were compared against African American predictive Global Lung Function Initiative (GLI) 2012 equations.^21^ The main spirometric parameters recorded were the forced expiratory volume in one second (FEV_1_), the forced vital capacity (FVC) and the ratio (FEV_1_/FVC). As per GOLD guidelines, post-BD airflow obstruction (FEV_1_/FVC < 0.7) was categorised into mild (FEV_1_ ≥80% predicted), moderate (50% ≤ FEV_1_ < 80% predicted), severe (30% ≤ FEV_1_ < 50% predicted) and very severe (FEV_1_<30% predicted).^20,21^

Normal spirometry was defined as FEV_1_ and FVC >80% predicted with FEV_1_/FVC>0.7. A restrictive pattern was defined as FVC <80% predicted with FEV_1_/FVC >0.7.^21^

### Home air quality monitoring

Twenty-four-hour home measurements of particulate matter 2.5 µg/m^3^ (PM_2.5_) were performed with RTI MicroPEM™ (RTI MicroPEM; Diamond Bar, CA, USA) (Cotonou and Douala) and PurpleAir-II-SD (Fajara and Kanifing). Gravimetric calibration for quality control was conducted monthly for the RTI MicroPEM, and two-weekly routine maintenance for the PurpleAir-II-SD (PurpleAir; Draper, UT, USA) sensor.^22^ Carbon monoxide (CO) measurement was performed with Lascar^®^ (Lascar Electronics, Whiteparish, England, UK) loggers. Measurement of the air pollution biomarker: exhaled carbon monoxide (eCO) was with a pre-calibrated CO check plus device. The latter is a short-term (<2 h) biomarker of exposure both outdoor and indoor air pollution exposure.^23^

Second-hand smoke exposure was assessed with the following questions: “Does anyone smoke cigarettes or tobacco inside the building where you sleep (do not include yourself)?” and “Does anyone smoke cigarettes or tobacco inside any building where you work or spend other time?” and “Are you ever exposed to smoke from burning refuse (waste, rubbish)?”. Traffic exposure was assessed with: “How many hours of your average day do you spend on the roadside of a major road? (in hours)” and “How many hours of your average day do you spend travelling on a main road? (include walking)”. Also, work exposure was assessed with: “In your job, do you breathe in vapours, dusts, gases or fumes for more than 15 h per week?” Finally, Crop cultivation and pesticide use were assessed with: “Do you cultivate crops?” and “Do you use pesticides in cultivation of your plants?”

### Statistics

A sample of 50 patients was planned from each centre based on feasibility, and timeline to get sufficient preliminary data as a prelude to larger, more definitive studies.

The primary exposure variables are home air pollution exposure levels: PM_2.5_ in µg/m^3^, CO in parts per million (ppm) and exhaled CO (in ppm and percentage of carboxyhaemoglobin). Values were presented as median with interquartile ranges (IQRs).

Secondary exposure variables included: exposures at home/daily life, traffic and occupational exposure. Primary outcome is the presence of CAD. Secondary outcomes are disease type based on spirometry test (e.g., chronic airflow obstruction, restriction etc), measure of disease control/ impact of control (ACT, CAT).^18,19^

Between-group comparison were performed using χ^2^, Fisher’s Exact test, *t*-tests or analysis of variance, as appropriate; *P* < 0.05 was considered statistically significant. Analyses was performed with R statistical package (R Computing, Vienna, Austria).

### Ethical considerations

The study protocol received ethical approval from relevant National Ethics Committees of the three countries: Benin (Universite De Parakou, Cotonou, Benin; Ref:0091/CLERB-UP/P/SP/R/SA), Cameroon (University of Douala, Douala, Cameroon; No:1632IEC-UD/06/2018/R), The Gambia (Gambian Government/MRC Joint Ethics Committee, Banjul, The Gambia; Ref 19133) and the Liverpool School of Tropical Medicine Research Ethics Committee (Liverpool, UK; LSTM REC 18-051 and LSTM REC 19-098). All participants provided written informed consent.

## RESULTS

In total, 98 patients (Benin, *n* = 35; Cameroon, *n* = 13; and The Gambia, *n* = 50) participated in this study.

### Sociodemographic characteristics of study participants

Benin patients were the oldest and patients in Cameroon were the youngest. Most patients lived in cities. Most Gambian patients were in employment (70.0%), while Benin patients were the least likely employed (29.0%). The rates of ever smoking were similar between the three countries (Table 1).

**Table 1.** Socio-demographic characteristics of study participants.

Characteristics	Benin	Cameroon	Gambia	*P*-value
	(*n* = 35)	(*n* = 13)	(*n* = 50)	
	*n* (%)	*n* (%)	*n* (%)	
Male sex	23 (66.0)	8 (62.0)	25 (50.0)	0.144
Age, years				
20–34	1 (4.0)	4 (33.0)	8 (16.0)	0.015*
35–49	5 (23.0)	3 (25.0)	16 (32.0)	
50–64	11 (50.0)	1 (8.0)	7 (14.0)	
65–79	5 (23.0)	4 (33.0)	19 (38.0)	
Median [IQR]	53.0 (43.0–63.0 )	48.0 (22.0–67.0)	51.5 (41.0–67.0)	
Current place of abode				
Town	25 (71.0)	9 (69.0)	49 (98.0)	0.170
Suburban	10 (29.0)	4 (31.0)	1 (2.0)	
Current employment status				
Employed	10 (29.0)	6 (46.0)	35 (70.0)	<0.001*
Ever reported smoking history	6 (17.0)	3 (23.0)	10 (20.0)	0.888
Work type				
Professional	2 (18.1)	1 (14.3)	2 (13.3)	0.147
Technical	3 (27.3)	2 (28.6)	0	
Trading	3 (27.3)	2 (28.6)	11 (73.3)	
Factory worker	3 (27.3)	2 (28.6)	1 (6.7)	
Transportation	0	0	2 (13.3)	
Work involves particulate matter exposure	9 (56.0)	5 (71.0)	13 (26.0)	0.013*
Passive smoking	5 (14)	5 (38)	13 (26.0)	0.178
Traffic exposure, h				
<1	23 (66.0)	6 (46.0)	48 (96.0)	<0.001*
>1	12 (34.0)	7 (54.0)	2 (4.0)	
Crop cultivation	4 (11.0)	1 (8.0)	6 (12.0)	0.900
Pesticide use	3 (9.0)	1 (8.0)	4 (8.0)	0.993

* χ^2^ with Yates correction.

IQR = interquartile range.

Workplace particulate matter exposure was highest in Cameroon (71.0%) and lowest in Gambia (26.0%). Reported passive smoking exposure was 38.0% in Cameroon, 26.0% in Gambia and 14.0% in Benin; 54% of the Cameroonian cohort spent more than one hour in traffic daily, while only 4.0% of Gambian participants reported traffic exposure greater than 1 h (Table 1). Aerosol use in the home was reported by respectively 16 (46.0%), 10 (77.0%) and 18 (36.0%) of the respondents in Benin, Cameroon and Gambia.

### Clinical characteristics of study participants

The most frequently reported symptom across all sites was wheeze (55.0%); shortness of breath was least reported (30.0%); 42% of patients reported cough. Asthma was the most common physician diagnosis across all sites (80%), followed by COPD (16.0%), and then chronic bronchitis (4.0%). Comparatively, Benin had the highest proportion of people with asthma (83.0%), followed by Gambia (80.0%). Cameroon had the highest proportion of people with COPD (23.1%), followed by Gambia (18.0%). Over half of the cohort had at least one exacerbation in the preceding year. In the previous year, ≥1 exacerbation was reported by 79.5% of the people with asthma and 68.7% of COPD patients, with no statistical difference in the rates of asthma exacerbation (*P* = 0.255) and COPD exacerbation (*P* = 0.519) across all sites. Furthermore, about two-third (66.0%) of the people with asthma in this cohort have poor disease control (ACT score ≤19). In 91.0% of those with COPD, the disease had at least a moderate impact on their lives.

Benin patients had the lowest mean FEV_1_ (66.5, standard deviation [SD] ±22.2), while Gambia reported the highest mean FEV_1_ (71.2, SD ±20.2). FVC followed a similar pattern (Table 2). There were no statistical differences in the FEV_1_, FVC and FEV_1_/FVC ratio in the three countries. (*P* = 0.707, *P* = 0.134 and *P* = 0.196, respectively). Prevalence of airflow obstruction was respectively 77.1%, 54.0% and 64.0% in Benin, Cameroon and Gambia (Tables 2 and 3).

**Table 2.** Clinical characteristics of study participants.

Characteristics	All	Benin	Cameroon	Gambia	*P*-value
	*n* (%)	*n* (%)	*n* (%)	*n* (%)	
Symptoms					
Cough	41 (42.0)	14 (40.0)	7 (54.0)	20 (40.0)	0.645
Current wheeze	54 (55.0)	20 (57.0)	10 (77.0)	24 (48.0)	0.153
Shortness of breath	29 (30.0)	6 (17.0)	2 (15.0)	21 (42.0)	0.028*
Physician diagnosis					
Asthma	78 (80.0)	29 (83.0)	9 (69.0)	40 (80.0)	0.669
COPD	16 (16.0)	4 (11.0)	3 (23.0)	9 (18.0)	
Chronic bronchitis	4 (4.0)	2 (6.0)	1 (8.0)	1 (2.0)	
Reported exacerbation within last 1 year					
0	47 (48.0)	15 (43.0)	3 (23.0)	29 (58.0)	0.076
1–2	16 (16.0)	4 (11.0)	3 (23.0)	9 (18.0)	
3–5	18 (18.0)	6 (17.0)	5 (38.0)	7 (14.0)	
>5	17 (18.0)	10 (29.0)	2 (16.0)	5 (10.0)	
Level of disease control/disease impact					
ACT					
Good (≥20)	26 (34.0)	6 (23.0)	5 (50.0)	15 (38.0)	0.255
Poor (≤19)	50 (66.0)	20 (77.0)	5 (50.0)	25 (62.0)	
CAT					
Mild (<10)	2 (10.0)	1 (12.5)	0 (0)	1 (10.0)	0.519
Moderate(10–20)	13 (62.00)	6 (75.0)	1 (33.0)	6 (60.0)	
Severe (≥21)	6 (28.0)	1 (12.5)	2 (67.0)	3 (30.0)	

Spirometry characteristics	All	Benin	Cameroon	Gambia	*P*-value
	(*n* = 98)	(*n* = 35)	(*n* = 13)	(*n* = 50)	
Spirometry indices, mean ± SD					
FEV_1_ (% predicted)	69.7 ± 21.5	66.5 ± 22.2	69.5 ± 25.4	71.2 ± 20.2	0.707
FVC (% predicted)	83.6 ± 17.7	78.1 ± 16.9	81.7 ± 23.6	86.8 ± 15.6	0.134
FEV_1_/FVC ratio	66.5 ± 15.4	63.9 ± 14.6	72.9 ± 14.7	66.7 ± 16.0	0.196
Spirometry patterns					
Normal	29 (29.6)	8 (22.9)	6 (46.0)	15 (30.0)	0.277
Airflow obstruction	66 (67.3)	27 (77.1)	7 (54.0)	32 (64.0)	
Restrictive	3 (3.1)	0	0	3 (6.0)	

* χ^2^ with Yates correction.

COPD = chronic obstructive pulmonary disease; ACT = Asthma Control Test; CAT = COPD Assessment Test; SD = standard deviation; FEV_1_ = forced expiratory volume in first second; FVC = forced vital capacity.

**Table 3.** Disease exacerbation, disease control and spirometry pattern by respondents’ diagnoses among all cohorts across the sites.

Characteristics	All	Asthma	COPD	Chronic bronchitis	*P*-value
	(*n* = 98)	(*n* = 78)	(*n* = 16)	(*n* = 4)	
	*n* (%)	*n* (%)	*n* (%)	*n* (%)	
Exacerbations					
0	22 (22.4)	16 (20.5)	5 (31.3)	1 (25.0)	0.756
1–2	53 (54.1)	43 (55.1)	9 (56.3)	1 (25.0)	—
3–5	11 (11.2)	9 (11.5)	1 (6.2)	1 (25.0)	—
>5	12 (12.2)	10 (12.9)	1 (6.2)	1 (25.0)	—
ACT^*^					
Good (≥20)	26 (34.0)	26 (34.0)	—	—	—
Poor (≤19)	50 (66.0)	50 (66.0)	—	—	—
CAT					
Mild (<10)	1 (9.1)	—	1 (9.1)	—	—
Moderate (10–20)	7 (64)	—	7 (64)	—	—
Severe (≥ 21)	3 (26.9)	—	3 (26.9)	—	—
FEV_1_^*^					
<80%	64 (66.7)	50 (52.1)	12 (75.0)	2 (50.0)	0.599
≥80%	32 (33.3)	26 (47.9)	4 (25.0)	2 (50.0)	—
FVC^*^					
<80%	41 (42.7)	30 (31.3)	9 (56.3)	2 (50.0)	0.447
≥80%	55 (52.3)	46 (68.7)	7 (43.7)	2 (50.0)	—
FEV_1_/FVC ratio^*^					
<70%	55 (57.3)	44 (58.0)	9 (56.0)	2 (50.0)	0.949
≥70%	41 (42.7)	32 (42.0)	7 (44.0)	2 (50.0)	—
Severity of airflow obstruction^*^					
FEV_1_ <30%	6 (6.3)	4 (5.3)	2 (12.5)	0	0.207
FEV_1_ 30–49%	10 (10.4)	5 (6.6)	4 (25.0)	1 (25.0)	—
FEV_1_ 50–79%	48 (50.0)	41 (54.0)	6 (37.5)	1 (25.0)	—
FEV_1_ ≥80%	32 (33.3)	26 (34.1)	4 (25.0)	2 (50.0)	—

* Two missing data among people with asthma.

COPD = chronic obstructive pulmonary disease; ACT = Asthma Control Test; CAT = COPD Assessment Test; FEV_1_ = forced expiratory volume in first second; FVC = forced vital capacity.

### Disease exacerbation, disease control and spirometry patterns by respondents’ diagnoses among all cohorts across the sites

About half (52.1%) of the patients with asthma and three-quarters (75.0%) of the obstructive airway disease patients had FEV_1_ <80% predicted. Nearly 60% of respondents with physician diagnosis of asthma fulfilled the GOLD definition of COPD, whereas 44.0% of those with diagnosed COPD did not meet the GOLD spirometric definition of COPD of FEV_1_/FVC<0.7 (Table 3).

### Air pollution exposure among respondents in the three countries

Home PM_2.5_ exposure was highest among the participants in the Benin (13.0 µg/m^3^, IQR 6.0–139.3)) and lowest among the Gambian respondents (4.4 µg/m^3^, IQR 1.7–11.1), with statistically significant difference at *P* < 0.001. Home CO exposure was highest in the CAD patients from Benin (1.6 ppm, IQR 0.9–2.3)), and lowest among those from The Gambia (0.4 ppm, IQR 0.1–0.6)). There was no statistically significant difference. Exhaled CO was highest in Benin (5.0 ppm, SD ±4.2) and lowest in Cameroon (1.9 ppm, SD ±0.8) (Table 4).

**Table 4.** Air pollution exposure among respondents in the three countries.

Characteristics	All	Benin	Cameroon	Gambia	*P*-value
	(*n* = 98)	(*n* = 35)	(*n* = 13)	(*n* = 50)	
	Median [IQR]	Median [IQR]	Median [IQR]	Median [IQR]	
Home PM_2.5_, µg/m^3^	6.0 [2.0–12.0]	13.0 [6.0–139.3]	5.0 [3.0–5.6]	4.4 [1.7–11.1]	<0.001
Home CO exposure, ppm	0.6 [0.2–1.6]	1.6 [0.9–2.3]	0.3 [0.1–0.35]	0.4 [0.1–0.6]	0.164
eCO, ppm, mean ± SD	0.6 ± 0.4	5.0 ± 4.2	1.9 ± 0.8	3.1 ± 1.5	0.217
Home PM_2.5_ in patients with asthma	6.0 [2.6–11.9]	10.0 [6.0–110.0]	5.0 [3.5–6.7]	4.3 [1.5–8.9]	<0.001
Home PM_2.5_ in patients with COPD, µg/m^3^	5.0 [2.0–16.7]	12.5 [6.0–116.5	3.5 [2.0–6.8]	4.2 [2.5–16.7]	0.197
Home PM_2.5_ in patients with chronic bronchitis, µg/m^3^	0 [0–3.0]	0 [0]	—	11.9 [0]	—
Home CO in patients with asthma, ppm	0.6 [0.2–1.6]	1.4 [0.8–2.1]	0.2 [0.1–0.4]	0.4 [0.1–0.6]	0.173
Home CO in patients with COPD, ppm	0.4 [0.3–1.7]	1.9 [1.6–2.8]	0.3 [0.27–0.8]	0.4 [0.2–0.7]	0.105
Home CO in patients with chronic bronchitis, ppm	2.3 [1.7–4.8]	2.0 [1.6–2.3]	10.9 [10.9–10.9]	1.9 [1.9–1.9]	0.135

IQR = interquartile range; PM_2.5_ = particulate matter 2.5*µ*m; CO = carbon monoxide; ppm = parts per million; eCO = exhaled CO; SD = standard deviation; COPD = chronic obstructive pulmonary disease.

## DISCUSSION

This is, to the best of our knowledge, the first study comparing non-communicable airway disease and air pollution exposure in multiple West African countries. In this cross-sectional study, we found that most patients living with CAD attending these city-based clinics are usually males, older than 50 years, residing in urban settlements, with up to three-quarters being symptomatic: cough, shortness of breath and wheeze. Asthma was the most diagnosed CAD across the countries, followed by COPD.

A notable finding of this study is that 58.0% of those diagnosed with asthma fulfilled the spirometric criteria for COPD, while 44.0% of those with physician diagnosis of COPD do not meet the spirometric criteria for COPD. This finding is similar to that reported by Binegdie et al. in their study of 519 chronic respiratory disease patients in outpatient clinics in Kenya, Ethiopia and Sudan. They found that 39.0% of clinician-diagnosed asthma patients fulfilled the criteria for COPD. Furthermore, despite the physician diagnosis of COPD in 7.9% of the patients, spirometry consistent with COPD was present in 35.0%.^24^ Possible explanation for these findings include: chronic airway remodelling in asthma and asthma overdiagnosis due to misdiagnosis of COPD as asthma because of reliance on clinical features in the absence of spirometry. Similarly, COPD underdiagnosis is a consequence of lack of spirometry and reliance on clinical suspicion. Clinical diagnosis without spirometry is not reliable in establishing chronic airway disease.

The overall prevalence of physician-diagnosed airway obstruction in this study was 100%. The overall spirometry confirmed prevalence of airway obstruction was 67.3%. This highlights the tendency of clinical diagnosis to over-estimate obstructive lung disease. The place of spirometry in establishing the diagnosis of chronic obstructive airway disease cannot be over-emphasised.^25^

The Gambia had majorly mild and moderate CAD severity, while Benin and Cameroon reported severe and very severe CAD in their respondents. Across all the sites, 11.8% of asthma patients and 38.0% of COPD patients have at least severe airway obstruction. About two-third (66.0%) of the asthma patients had poor disease control (ACT score ≤19). In 91.0% of those with COPD, the disease had at least a moderate effect on their lives. The finding of poor disease control also featured in a North African study of over 7,000 eligible people with asthma, where only 16.0% were found to have good asthma control (mean ACT score of 21.8).^26,27^ In addition, a study of 449 adults with asthma in Uganda by Kirenga et al. highlighted the fact that 28.1% (*n* = 126) were uncontrolled with an ACT score of <15.^28^

We found the between country differences in PM_2.5_ median exposure among asthmatic respondents was statistically significant (*P* < 0.001), while PM_2.5_ of COPD patients and CO exposure of asthma patients were statistically insignificant (*P* = 0.197 and *P* = 0.173, respectively). Also, there was no significant association between air pollution and disease exacerbation, asthma control and the impact of COPD on patients’ lives. In the absence of very similar studies in sub-Saharan Africa, Dionisio et al. reported fixed site household mean PM_2.5_ of 275 µg/m^2^ and 219 µg/m^2^ for the mothers and the children in their study in Gambia.^29^ The CO averages were respectively 2.4 ppm and 1.5 ppm in mothers and children. This study and ours were both urban in location but the latter did a 48-hour measurement while we measured for 24 hours.^30^ In addition, there was a 13-year difference which would likely affect the energy source used at home then as compared with now.

The proportion of ever smoking tobacco, passive smoking and home aerosol use (atomised spray chemicals such as mosquito insecticides and air fresheners) were highest in Cameroon (23.0%, 38.0% and 77.0%, respectively). Ever smoking was 23.0% or less at each site among the respondents. Most of the respondents spend significant time in and around their homes as they are mainly traders and technicians, thus exposing them to locally used aerosols and fumes from the mostly biomass-powered kitchen which is usually near the house.^29,31^ The study by Binegdie et al. in East Africa reported that 83.0% of the respondents never smoked but 19.0% and 32.0% were exposed to passive smoking at home and at work.^24^

Our study was not without limitations. The total number of respondents were less than 100, thus affecting the power of the study and limiting the generalisability to the cohort studied. In addition, the instruments used in The Gambia differed from those used in Benin and Cameroon: PurpleAir-II-SD and Lascar CO devices in Gambia and, RTI MicroPEM and Lascar CO devices at the other two locations. This device difference between the sites can affect data comparability. However, RTI MicroPEM uses both gravimetric and optical laser methods to measure fine particulate matter while PurpleAir uses optical laser method, thus providing some methodical overlap for the two equipments. We recognise the latter as a significant limitation of our study.

The study had some strengths: it was a multi-country, multi-locational study in an environment with paucity of information on CAD, thus allowing for inter-country comparisons. Furthermore, PurpleAir-II-SD outside/ambient device was used for home/indoor air pollution measurement in our Gambian cohort, a use to which it is not usually put to. We had the confidence to apply this innovation because the equipment has been shown to perform consistently and accurately at very high and very low particulate matter exposure.^32^ This has added another affordable tool for air quality researchers to measure indoor or home PM_2.5_ exposure measurement, especially in resource-constrained settings like sub-Saharan Africa.^22^

## CONCLUSION

In conclusion, in patients attending these city-based chest clinics, asthma is the most common diagnosed CAD, with many participants having cough, shortness of breath and wheeze mainly. Nearly half of those with clinical diagnosis of COPD did not fulfil the spirometric criteria for this, pointing to the unreliability of clinical diagnosis in CAD management. Most of them have poor disease control, passive smoking exposure, and home PM_2.5_ and CO exposure. Concerted efforts are necessary to scale up spirometry, better case management, provision of clean fuel sources and improved air quality.
